# Towards a Fog-Enabled Intelligent Transportation System to Reduce Traffic Jam

**DOI:** 10.3390/s19183916

**Published:** 2019-09-11

**Authors:** Celso A. R. L. Brennand, Geraldo P. Rocha Filho, Guilherme Maia, Felipe Cunha, Daniel L. Guidoni, Leandro A. Villas

**Affiliations:** 1 Federal Rural University of Pernambuco (UFRPE), UAST, Gregorio Ferraz Nogueira Av., Serra Talhada, Pernambuco 56909–535, Brazil; 2 Institute of Computing, University of Campinas (UNICAMP), 1251 Albert Einstein Av., Campinas SP 13083, Brazil; 3 Department of Computer Science, University of Brasília, Distrito Federal 70000-000, Brazil; 4 Department of Computer Science, Federal University of Minas Gerais, Belo Horizonte 31270-901, Brazil; 5 Department of Computer Science, Pontifical Catholic University of Minas Gerais (PUC Minas), Belo Horizonte 30535–901, Brazil; 6 Department of Computer Science, Federal University of São João del-Rei, São João del-Rei 36301-360, Brazil

**Keywords:** vehicular networks, fog computing, intelligent transport system, mobile edge computing

## Abstract

Frustrations, monetary losses, lost time, high fuel consumption and CO${}_{2}$ emissions are some of the problems caused by traffic jams in urban centers. In an attempt to solve this problem, this article proposes a traffic service to control congestion, named FOXS–Fast Offset Xpath Service. FOXS aims to reduce the problems generated by a traffic jam in a distributed way through roads classification and the suggestion of new routes to vehicles. Unlike the related works, FOXS is modeled using the Fog computing paradigm. Therefore, it is possible to take advantage of the inherent aspects of this paradigm, such as low latency, processing load balancing, scalability, geographical correlation and the reduction of bandwidth usage. In order to validate FOXS, our performance evaluation considers two realistic urban scenarios with different characteristics. When compared with related works, FOXS shows a reduction in stop time by up to 70%, the CO${}_{2}$ emissions by up to 29% and, the planning time index by up to 49%. When considering communication evaluation metrics, FOXS reaches a better result than other solutions on the packet collisions metric (up to 11.5%) and on the application delay metric (up to 30%).

## 1. Introduction

The unplanned development of urban centers often is associated with severe socio-economic problems. Such uncontrolled urban growth typically causes significant stress on city structures due to the unexpected demand of various resources and services. One of the most affected sectors is the urban transport systems, in which inefficiencies may lead to many negative consequences. Among them are the increase in greenhouse gas emissions and many hours stuck in traffic congestions, thus resulting in health issues and monetary losses. For instance, the congestion cost in the United States, the United Kingdom and Germany were almost $461 billion in 2017 [1].

One approach to prevent these problems is the development of an Intelligent Transport System (ITS). An ITS uses communication, processing and sensing technologies to improve the urban traffic and consequently the flow of vehicles in the urban road. Moreover, an ITS does not only aim to provide traffic management services (for instance, to prevent traffic jam) but also security management services and infotainment applications to drivers, passengers and pedestrians [2,3,4,5,6]. In ITS, vehicles are equipped with sensors (e.g., GPS and Galileo), processors and wireless communication modules. In this way, vehicles can communicate with other vehicles through vehicle-to-vehicle (V2V) communication and with the network infrastructure (e.g., RSU—Road Side Unit) through vehicle-to-infrastructure (V2I) communication.

ITS services and applications have intrinsic characteristics regarding the way they process, store and disseminate a vast amount of data generated in ITS [7,8,9]. Theses characteristics imply in some issues for ITS services requirements such as mobility, frequent network disconnections, networking latency and end-to-end responsiveness time. Thus, designing ITS services that have a required quality of service (QoS) is a challenge [8,9,10].

The main benefits to designing an ITS with Fog paradigm are [11]: *low latency*–some ITS data have strict time constraints, such as data for re-route systems; *predominant wireless access*–modern ITS systems heavily rely on wireless communications; *wide geographical distribution*–ITS has sensors geographically spread. However, the scope of the data gathered is restricted to the location of the sensors that generated such data; *real-time interaction*–re-routing systems have real-time requirements; *mobility*–an ITS is used to optimize the mobility of vehicles in the city. Although, the ITS may also leverage mobility to perform data delivery activities to various stakeholders; *scalability*–an ITS needs to be scalable, due to the high number of vehicles and sensors; and *extensibility*–if the city grows, the ITS infrastructure also needs to grow to support the expanded region. These characteristics enable Fog Computing to offer an ideal platform for highly dynamic and heterogeneous ITS environment [9].

Generally, route suggestion services rely on data from specific regions, as traffic conditions, which may be irrelevant to other regions of a city. In this scenario, such service may exchange a large amount of data from heterogeneous data sources [7,12] to monitor the traffic conditions in a particular region. Moreover, the data may have real-time constraints and it can be disseminated using different communication technologies [13] and considering the dynamic topology, frequent network disconnections and cooperative communication [14]. It is worth noticing that sending data to a single central entity (e.g., Cloud) is a waste of system resources, such as network bandwidth. Moreover, data transmissions are more vulnerable to specific problems, such as delays, data loss, scalability, and communication disruption. Hence, route suggestion services in ITSs are not well suited to centralized architectures such as Cloud computing [8,12,13,15,16,17].

**Figure 1:**
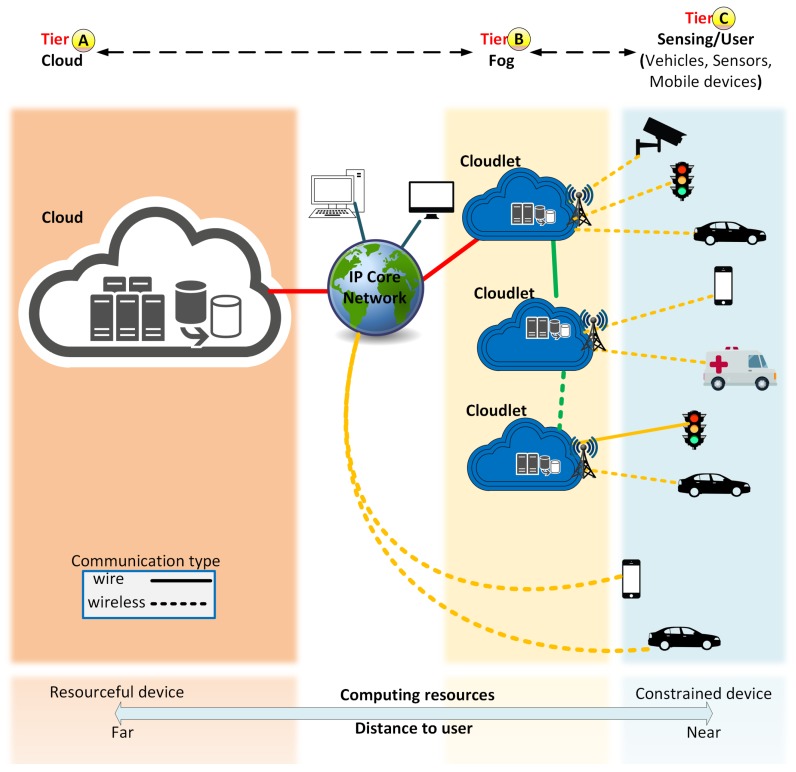
Cloud and Fog representation.

In this scenario, a route management service that takes advantage of the features of the Fog computing paradigm is extremely desirable in ITS. This happens because the Fog computing paradigm moves its resources (storage and processing) to the edge of the network, thus bringing the available resources as close as possible to end-users without the assistance of the Internet [11]. The Fog computing paradigm is based on entities named *Cloudlet* which have processing and communication capabilities (e.g., micro-data centers) and are geographically distributed to be closer to the access networks [18]. Since Cloudlets resources are closer to the end devices, they allow a faster response time and a local service decision. Thus, the Fog paradigm provides geo-computation and faster and less costly communication when compared to a Cloud. Although the Fog paradigm has lower computing capacity when compared with the Cloud, they can use the Cloud data centers whenever necessary. This approach forms a multi-tier architecture (see Figure 1), which is hierarchically organized with varying types of capabilities and end-user proximity. Cloud computing, represented in Figure 1 Tier **A**, possesses a more powerful resource, however the longer distance to retrieve data and the presence users beyond congested connections due to the use of the Internet often limit the real-time services and increase the network cost, especially considering a high dynamic vehicular network topology. Fog paradigm and Cloudlets environment are shown in Figure 1 Tier **B**, where the resources are closer to the end devices permitting a faster response time and a local service decision. Finally, users and sensors devices (e.g., vehicles, road sensors, cameras) are represented in Figure 1 Tier **C**.

In the literature, several studies address the problem of route management in urban centers [19,20,21,22,23,24,25,26]. In most of them, this kind of service employs an architecture for carrying out the monitoring and traffic control that relies on information about the vehicles, as well as the characteristics of the routes. However, these architectures also have to exchange, process and store a considerable amount of data generated by the devices that are embedded in the vehicles and that are used for monitoring the city traffic. Thus, problems related to processing (e.g., load balance, response time) and data transmissions (e.g., delays, data loss and communication disruption) become a concern. Besides, in route suggestion services, the response time to perform the decision-making process must be within an acceptable time frame so that the information is still useful in order to the vehicle’s driver to carry out the necessary route changes.

Given the aforementioned limitations, this article proposes a traffic service to control congestion, named FOXS–***F**ast **O**ffset **X**path **S**ervice*, which is based on the Fog computing paradigm. FOXS uses the Cloudlets to monitor the traffic conditions and to calculate the vehicle route. In this way, FOXS allows that the computational power resides closer to where it is most required, thus dividing the system load and increasing the overall scalability of the system and holding the capability to collect, process and store large volumes of data. For that, FOXS uses the network infrastructure Road side Units (RSUs) as a Cloudlet entity, which is deployed in the city to manage the traffic of vehicles. For its operation, a mechanism that gathers all necessary data from vehicles and road sensors was developed. Such mechanism optimizes the delivery rate and reduces the number of messages in the system. Then with the data collected by the corresponding Fog entities, the level of congestion of the roads is estimated. Finally, according to the conditions of the roads, the corresponding Fog entities calculates a new route as a suggestion.

This article was based on our previous work [27]. It should be stressed that through an analysis of the base work, we improved and developed new methodologies and techniques to make FOXS more efficient. Some of the improvements are the increase in the message delivery rate, the reduction of the number of necessary RSU to fully cover the scenario and the traffic classification for better suggestions of routes to vehicles.

In addition, we extend the previous experiments proposed in Reference [27]. Other routes services proposed in the literature were implemented and compared with FOXS. The results obtained, evaluated in different and realistic urban scenarios, demonstrate that the use of congestion control services can reduce losses due to the traffic jam. In particular, FOXS reduced the stop time by up to 70 %, the planning time index by up to 49 % and, CO${}_{2}$ emissions by up to 29 %. The network also improved with a reduction of the packet collisions (up to 11.5 %) and the application delay (up to 30 %).

The remainder of this article is structured as follows. Section 2 presents an overview of the literature about approaches to minimize congestion in urban centers. Section 3 presents the FOXS traffic service and its design and components. Section 4 presents the performance evaluation of our proposal, along with the methodology used and the results. Finally, Section 5 discusses the conclusions and future work.

## 2. Related Work

This section presents related works that address the problem of route management, that is, traffic congestion management of vehicles in urban centers. In the last few years, such a problem has been explored by several works [19,20,21,22,23,24,25,26,28,29]. However, we have not found works that address such problem through the Fog computing paradigm to improve system performance, such as the load balancing (network, processing), the response time and the network load.

There are several works [30,31,32] that present methods for describing and predicting traffic behavior. Hussein et al. [30] proposed a model for predicting short-time traffic conditions using a neural network approach (time lagged recurrent neural network—TLRN). Reference [31,32] proposed a stochastic model that assists the prediction of traffic flow uncertainties (e.g., weather conditions, public holidays, special events). These models can be used as an additional feature for routing algorithms thus improving their efficiency. This would be an advantage for FOXS, since the Cloud could run this batch and thus inform the traffic forecast in each region separately for each RSU-Cloudlet.

The system proposed in Reference [21] is responsible for traffic monitoring and vehicle re-routing to decrease the traffic congestion of vehicles. The goal is to reduce the driver’s travel time, as well as the CO_2_ emissions and fuel consumption of vehicles. To this end, real-time data about vehicular traffic conditions, such as position, speed and direction are gathered by a centralized system through vehicle-to-infrastructure (V2I) communication. Four steps are periodically executed in the traffic care system: (a) *Data collection and representation*, which describes the network using a directed graph, in which the weights are the average travel time; (b) *Congestion prediction* is the service that periodically checks all road segments to detect signs of congestion; (c) *Selection of Vehicles to Be Rerouted* selects candidate vehicles near the congested roads; and (d) *Choose alternative routes* for each previously selected vehicle. The authors employ three strategies for calculating new routes: (i) Dynamic Shortest Path, which calculates the route with the lowest travel time; (ii) Random *k* Shortest Paths, which selects the *k* lowest travel time path routes and assign, at random, one of them to the vehicle; and (iii) Entropy Balanced *k* Shortest Paths, which is an enhancement of Random *k* Shortest Paths, in which it is considered the impact of the selected road on the future density of the road. However, these strategies have the following drawbacks: (i) congestion in other places due to the suggestion of routes to the same area; (ii) long routes can be selected to reduce the traffic of vehicles in another area; and (iii) the use a central server requires a substantial computational resource and network communication, so the use of a central server is not salable.

The work described in References [24,25] proposes services for real-time traffic management with route planning and congestion detection. CHIMERA [25] was based in SCORPION [24] and its main difference is in the route suggestion. In Reference [25], an intelligent traffic system was proposed which improves the overall spatial utilization of the road network to reduce the average vehicle travel costs, named CHIMERA. In CHIMERA, vehicles provide their information (ID, current position, route and destination) to an RSU entity through a single-hop long-range communication, such as 4G and LTE. For this, CHIMERA was modeled into three main parts: (i) congestion detection;(ii) traffic classification; and (iii) route suggestion. CHIMERA perform congestion detection and traffic classification using *K-NN* (k-nearest neighbors) according to the average speed and the density of the path. As output, it informs the road classification based on the traffic condition (e.g., free-flow, slightly congested, moderately congested and severely congested). Finally, CHIMERA uses the K-Shortest Path-based algorithm for the route choice. However, different from this article, these solutions [24,25] did not propose a message scheduling mechanism to reduce problems in data transmission, such as packet collision. Another problem, solved in this article, is that communication between RSUs is not implemented. Thus, the RSU is not aware of the traffic conditions in other regions of the map, thereby limiting the efficiency of the routing system.

Meneguette et al. [33] proposed a solution, named INCIDEnT (INtelligent protocol of CongestIon DETection), based on an Artificial Neural Network (ANN) to estimate congestion level and maximize the urban traffic flow. The ANN uses the average speed and the density of vehicles on the road as the input of the system to classify the traffic and suggest new routes for drivers. The congestion is classified in three levels: Free; Moderate and Congested. Finally, the classification data are disseminated by all vehicles on the road through periodic beacon messages. When a vehicle receives a message about a road congestion level, the ANN can decide whether to keep its current route or calculate an alternative route. However, the solution does not have full knowledge of the map neither a method to avoid the overlapping routes, which can in turn generate a new traffic jam. Another problem detected is that it does not implement any broadcast suppression mechanism, thus decreasing its efficiency, especially in a high-density scenario.

Doolan et al. [34] proposed a VANET (Veicular Ad Hoc Networks) routing solution—named EcoTrec—aimed to reduce the CO${}_{2}$ emission without significantly affecting the travel time. For this, each vehicle periodically disseminates data about its fuel consumption, current route and average road speed. Thus, EcoTrec determines the roads conditions and in a distributed way, each vehicle calculates a new route. To avoid the various vehicles always attribute the same best route, EcoTrec randomly assigns the second-best route to some vehicles. The EcoTrec architecture possesses three main parts: (i) VehicleModel with the vehicle and embedded sensors characteristics; (ii) a RoadModel with a road representation and characteristics that are allocated in the central server; and (iii) a TrafficModel with the traffic condition based on the VehicleModel and RoadModel characteristics. However, all vehicles in the system send messages to the neighboring vehicles and the central server to update the TrafficModel. Moreover, the vehicles compute their route based on the TrafficModel received from the server. Thus, the scalability of the system is compromised by a large number of messages exchanged.

Younes et al. [26,35,36] proposed ECODE (Efficient road Congestion Detection protocol) and the ICOD (intelligent path recommendation protocol), that uses V2V and V2I communications to detect traffic congestion on each road segment. ICOD [36] was based in ECODE [26,35], however it has a mechanism that enables users to choose which type of route the system will provide according to users’ concerns and priorities (e.g., fuel consumption, traveling time, road segment context). For that, RSUs are placed in every intersection and, using V2V, vehicles send an advertisement message (ADV) containing its information (e.g., ID, Speed, location, direction, destination and timestamp) to neighboring vehicles. When a vehicle receives an ADV, the information received is aggregated to the neighbor report table (NR) to calculate a traffic monitoring report (TMR) that informs the average road speed, the density and the estimated travel time. Furthermore, the closest vehicle to any RSU sends the TMR to that RSU. When the RSU receives the TMR, it checks its local information to determine the best direction for each destination, then it disseminates a RecomReport message. Finally, when a vehicle receives a RecomReport message, it changes its route towards the destination and forwards the message to the one-hop neighbors. Differently from FOXS, ECODE does not possess full knowledge of the map, thus causing the same problem in Reference [33], and despite it having a forward control message mechanism, the large number of necessary messages to perform its work causes scalability constraints as in Reference [34].

Wang et al. [37] proposed a solution called Next Road Rerouting (NRR) to alleviate urban traffic jam. To calculate a route, NRR applies a heuristic based on a cost function that uses information like road occupancy, travel time, distance to destination and the congested road. Vehicle routing is made in two steps. In the first step, the intelligent Traffic Light (iTL) module checks whether any of its intersection roads is congested. If congested, iTL sends beacons informing the vehicles about the congested road. Thus, vehicles that pass through this road request an alternative road to iTL. In the second step, when the vehicle receives the alternative road, this vehicle requests to central server a new route from the road suggested by iTL to the final destination. NRR has a 3-tier architecture with (i) Central Manager located at the Traffic Operation Center, (ii) intelligent Traffic Light (iTL) with loop detectors disposed on each intersection and (iii) local computers residing in the middle tier connected to iTL and Central Manager. However, this solution depends only on iTL to acquire road traffic information, thus being necessary the installation of iTL in all intersections. Also, NRR needs an Internet connection to work correctly, while this assumption is not necessary for FOXS.

Jeong et al. [38] proposed a cloud-based system for traffic optimization called Self-Adaptive Interactive Navigation Tool (SAINT). In this system, vehicles report the road traffic conditions to the traffic control center hosted in the Cloud. RSU and eNodeB (from cellular network) require Internet connections to communicate with the Cloud, thus vehicles are equipped with 802.11p and 4G. To reroute vehicles, SAINT uses a modified Dijkstra’s algorithm where the weight function takes into account the vehicle’s delay to reach the roads of the route. Thus, the probability of a route becoming very popular and causing a new congestion is reduced. However, such solution has some limitations. For instance, vehicles must continuously inform the conditions of the routes through the Internet connection to the Cloud. Another limitation is the Dedicated Short-Range Communications–DSRC standard communication used by vehicles since it does not have any mechanism to work correctly in high-density scenarios, such as urban centers.

Table 1 shows the features of the related works and highlights the main contributions of this article. It is observed that no work uses the Fog computing paradigm to improve system performance. Because of these features, we developed a routing service, named FOXS, that overcome the gaps of existing approaches. Therefore, FOXS uses a Fog computing paradigm allowing the cooperation of RSUs, distributing the network and processing capabilities.

## 3. FOXS–Fast Offset Xpath Service

**Figure 2:**
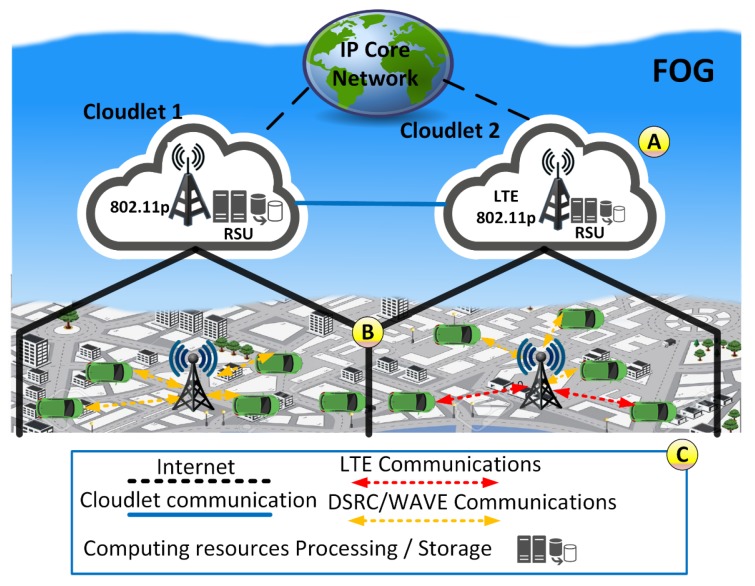
FOXS design.

This section presents a traffic management service for route suggestion: FOXS, Fast Offset Xpath Service. FOXS is based on the Fog computing paradigm, which allows distributing the computer and communication resources among ITS components using the various computational entities, as presented Figure 2.

The FOX is composed of two main components, Vehicles and Cloudlets. Vehicles have communication capabilities and embedded sensors (e.g., GPS) that are responsible for collecting data about road conditions as well as receive/request new routes. Cloudlets are implemented as RSU and the Cloudlet set forms the Fog computing environment (see Figure 2, Label A). Cloudlets are spread in the scenario according to the RSU communication range to reach full coverage of the entire map. Without loss of generality, the RSU deposition follows the cellular antennas deployment (hexagonal areas). Each Cloudlet is responsible for collecting, storing and analyzing the data (vehicles position and velocity, road occupancy, level of congestion) of a specific region and compute routes for vehicles in its region (see Figure 2, Label B). By considering specific areas, the data is kept closer to end-users (vehicles) and road sensors, resulting in a more efficient processing, communication and quick response time. As seen in Figure 1, the Cloudlet is in Tier B and Vehicles are in Tier C.

**Figure 3:**
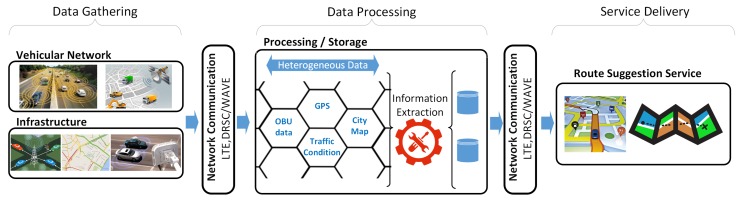
The data flow of the three steps of FOXS implementation design.

It is important to point out that the spatial context-aware characteristics allow that each Cloudlet to be independent of each other. To increase the flexibility and environment communication penetration, the Cloudlet can be equipped with various communication technologies (e.g., LTE, 802.11p) and multiple antennas (Figure 2, Label C). For a better understanding of the FOXS’s design, three steps are presented below, as shown in Figure 3: (i) Data Gathering, Section 3.1 describes the data gathering process, the algorithms and mechanisms developed for this role; (ii) Data Processing, Section 3.2 presents the data transformations and road traffic classification; and (iii) Service Delivery, Section 3.3 describes the algorithms and methods used to compute the new route and delivery it to the users.

### 3.1. Data Gathering and Communication

**Figure 4:**
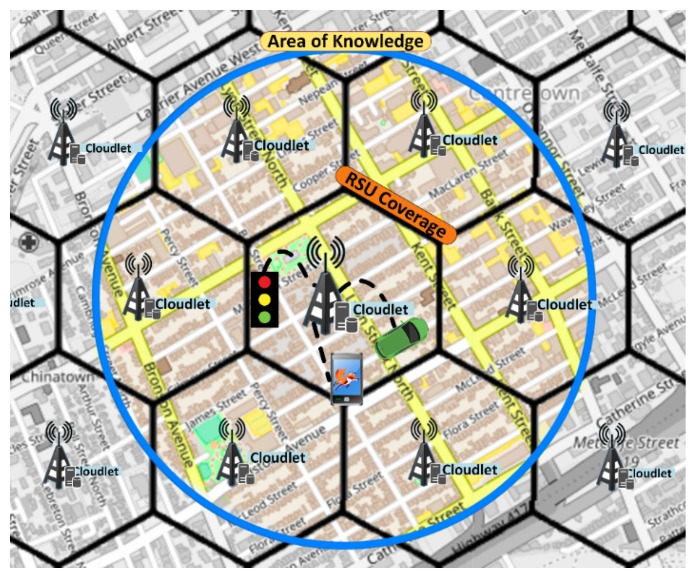
Cloudlet Division.

In order to perform Data Gathering, we need to deploy the RSU infrastructure, that is, the Cloudlets. After the deployment process, the Cloudlet gathers road/vehicle data.For this implementation, we use a set of RSU with Cloudlet capability spread homogeneously in the environment to achieve full coverage of the entire map (see Figure 4). Each Cloudlet is composed of a single RSU with communication capability to others RSUs and with vehicles. The communication between RSU is made by wire and RSU—Vehicle communication uses 802.11p wireless communication. Vehicles are equipped with GPS and On-Board Unit (OBU), which is a device mounted on vehicles that has processing power and allows DSRC communications with other OBUs or RSUs. Cloudlets are responsible for collecting all data generated inside its communication coverage (represented by hexagons in the Figure 4).

Cloudlets are distributed accordingly to their coverage by applying the Hexagonal Binning ([39]) algorithm to reach an efficient relationship between the number of RSUs versus city map size. This algorithm is based on cellular base-station deployment models [40]. This strategy is consistent with the Fog paradigm once the map is partitioned, sharing the users between each Cloudlet and the resources are brought closer to the users. The algorithm for Cloudlet distribution is based in the dimensions of the map and the Cloudlet coverage. So, the number of RSUs and its coordinates are assigned. If an RSU is assigned to a region that does not have any road, it will be removed.

For the service to work correctly, the components of the traffic management system, such as Cloudlets and vehicles, send control data periodically (beacons) informing about the roads traffic conditions in their region and other types of data that FOXS uses. The acquisition of this data is executed in a distributed way using the communication capability of the Cloudlets. Cloudlets send beacons informing its position, the route interval for that region and the list of roads congested inside its region. Vehicles use such information to find nearby Cloudlets to send traffic information and request a new route. The data sent are the vehicle speed, position, time spent to move on each road and its current route. These data are sent periodically through beacons to the closest Cloudlet. Once the Cloudlet receives data about a specific region, it uses with the proposal of acquiring knowledge to execute the traffic service. The data exchanged considering the proposed cloud-based architecture and the DSRC/WAVE communication protocol is illustrated in  Table 2. Our propose takes advantage of the DSRC/WAVE control and service channels to better use the wireless resources.

In order to improve the communication between vehicles and RSU, a mechanism for message scheduling was developed to increase the packet delivery rate in the architecture using a DSRC/WAVE communication standard. The mechanism (presented in Algorithm 1) has the objective of scheduling the time of sending the packets to the 802.11p MAC layer to avoid the resynchronization problem ([41]). The mechanism also schedules the messages to be sent according to their size and the node bandwidth (line 15, Algorithm 1). For this, there are two queues for the messages (lines 4 and 5), one to the control channel (CCH) used to send control messages as beacons and another to the service channels (SCH) used for all other messages in the service. Algorithm 1 verifies the channel type of the message (lines 7 to 10) then assigns the send delay time according to the last message transmitting time in the corresponding queue (line 15). If the queue is empty, a value of zero is assigned to the delay (line 12). Then an additional delay is calculated based on the active channel (lines 17 to 27). Thus, this mechanism ensures that when the network layer sends a message to the MAC layer, the message will be sent promptly.

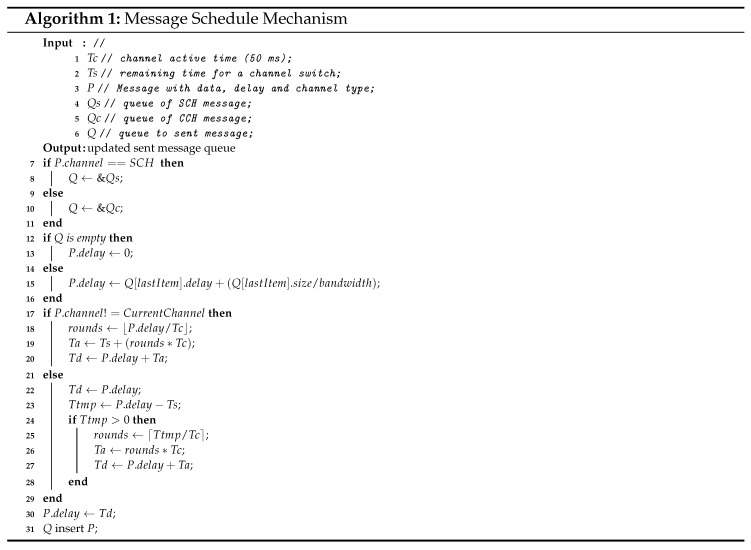



### 3.2. Data Processing

In this phase, the system processes the collected information from the previous step. Once the map is all covered by Cloudlets, each Cloudlet has the responsibility of collecting and processing only road data within its coverage. Thereby, limiting the data gathering/processing to a smaller region, reducing the cost of communication and making a processing load balance between Cloudlets in the system.

However, as described in Reference [4], the size of the region (amount of information) that will be used to compute the new route have an impact on the quality of the route. Therefore, a router region that is just the radio coverage of the Cloudlet may be too small for efficient routing. To solve this problem, the Cloudlet acquires information about roads that are in the coverage of other Cloudlets in order to improve the routing solution by increasing the amount of information (with roads and their features and current traffic situation) that the routing algorithm will use. This additional knowledge area contains information of roads that are under the responsibility of other Cloudlet and is called Area of Knowledge (AoK) (inside the blue circle in Figure 4). The Area of Knowledge is at least the size of the Cloudlet coverage area. The size of the AoK affects the performance of the service, since a larger AoK (e.g., more roads to route) results in a better result but the computation time is increased.

Each Cloudlet periodically updates the weight of each route based on information gathered by vehicles inside its coverage. A  multi-weight directed graph G=(V,E) is used to represent the AoK, where *V* is the set of intersections within range of the AoK (representing the vertices) and *E* is the set of roads connecting the intersections (representing the edges). The weight of each road $E_{i,j}$ of *G* where: *i* is the relation of the maximum allowed speed in the road inversely proportional to the speed at which vehicles travel on the road. Therefore, if the vehicle speed is close to the maximum speed allowed on the road, the weight of the road is lower; *j* is the road occupancy that is inferred through vehicle’s positions sent by vehicle beacons.

The periodic road weight updating is done in the following way. The Cloudlet makes averages of the data sent by vehicles that pass in a specific road for a time interval. Next, is used the Exponential Moving Average (EMA) to obtain the new road weight. The EMA is used to smooth out a large oscillation in the road weight that may occur toward recurrent events such as a vehicle parking or stopped for a short period of time. Thus, implying in an abrupt increase of the road weight implying on the road choice in the routing process.

The road weight *i* is used by the routing algorithm (described in the next section (Section 3.3)) and the weight *j* is used with the *i* to road classification. The road classification is used to inform vehicles which roads have sing of congestion so, request a new route based in this information. The road classification is based on Level-Of-Services (LOS) present in the Highway Capacity Manual (HCM) [42]. The HCM uses the speed and density of vehicles on the roads to measure the capacity and quality of traffic. The HCM classify the congestion into six levels between level A as free-flow to F as congested flow. Each LOS level defines the minimum and maximum speeds for each level based on the maximum speed allowed on the road and by maximum occupancy capacity. We consider a road congested when the road speed is classified as LOS C or the road occupation is classified as LOS D. These threshold levels were chosen because that is when road presents signs of emerging congestion. Thus, FOXS takes action in order to avoid the formation of congestion.

**Figure 5:**
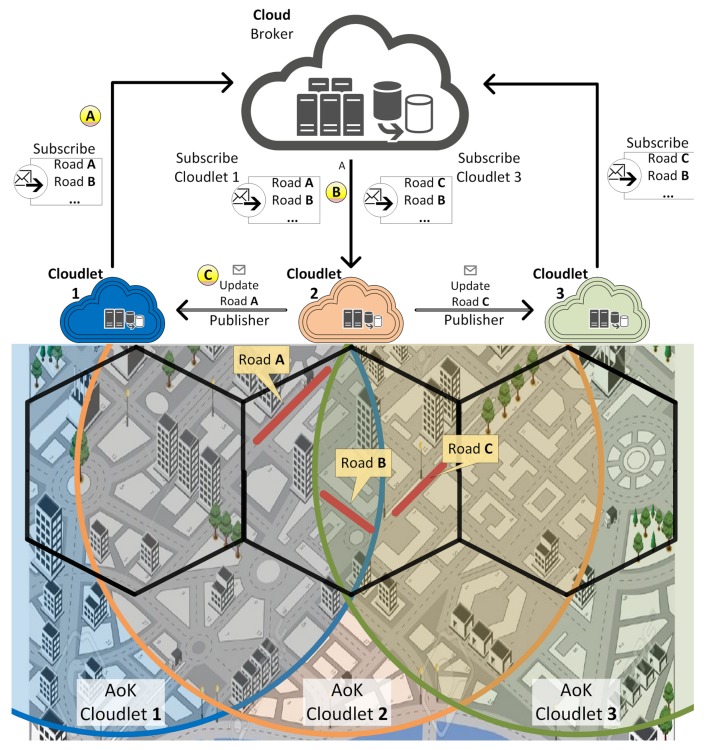
FOXS publish-subscribe protocol.

Note that each Cloudlet only classifies and updates the weight of the roads in its coverage area. To update roads on its AoK outside its coverage, Cloudlets share the road knowledge between them using publish/subscribe paradigm [43]. Subscribers register in events and asynchronously they are notified of events generated by publishers. The publish/subscribe protocol developed to FOXS has based on MQTT (Message Queuing Telemetry Transport) [44]. For that, all Cloudlets in the system are publishers, subscribers and the Cloud server is the broker (see Figure 5). In the beginning, each Cloudlet subscribes in the road update process by sending a road list including all roads inside its AoK except for roads inside its radio coverage (represented by hexagons). This list is sent to the Cloud server that has global knowledge of the map division (represented in Figure 5, Label A). After, the Cloud server notifies each Cloudlet (publisher) that is responsible for each road from the received list (see Figure 5, Label B). Cloudlets (publisher) notify all Cloudlets that are subscriber about road updates (see again Figure 5, Label C). Note that several Cloudlets may be intersected on the same path. The publish-subscribe method is an intesrting choice for ITS because of its asynchronous nature. The method also has the ability of work with context-aware applications, since interests flows considering a specific geographic region [45].

After, all these update processes, the Cloudlets disseminates in beacon messages the list of congested roads inside of its AoK. Thus, FOXS takes action in order to avoid the formation of congestion.

### 3.3. Service Delivery

In this phase, each Cloudlet performs the detection and control of congestion by calculating alternative routes to the vehicles. Thus, decreasing the load on the congested roads. Each vehicle, periodically, checks if it will pass through a congestion road. For that, the vehicles receive a beacon message sent by the Cloudlet with a list of congested roads. This list only contains roads belonging to the Cloudlet’s AoK.

**Figure 6:**
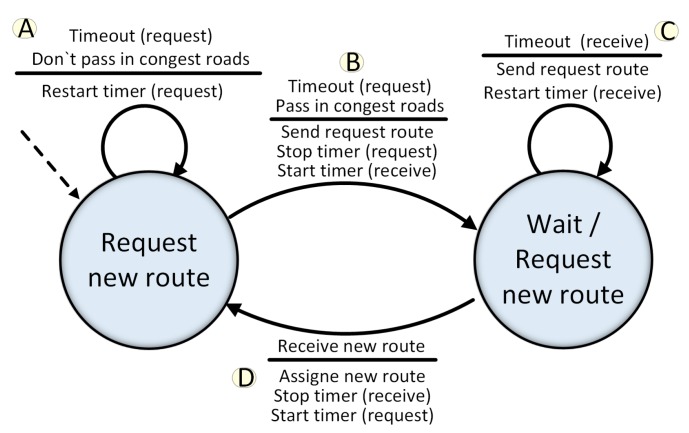
State diagram: Request new route.

Hence, at each route interval, the vehicle checks if its route passes through a congested road. The service delivery mechanism is illustrated in Figure 6. If it does not pass (Figure 6 stage A), the router interval is restarted. Otherwise, a message is sent to the closest Cloudlet requesting a new route and recovery time is started (Figure 6 stage B). The recovery time is a fault-tolerance mechanism that checks if the vehicle has received the requested route within a specific time interval. A new request message is sent in case of failure to receive (Figure 6 stage C). When the vehicle receives the new route, it is assigned and the route interval time is started (Figure 6 stage D).

**Figure 7:**
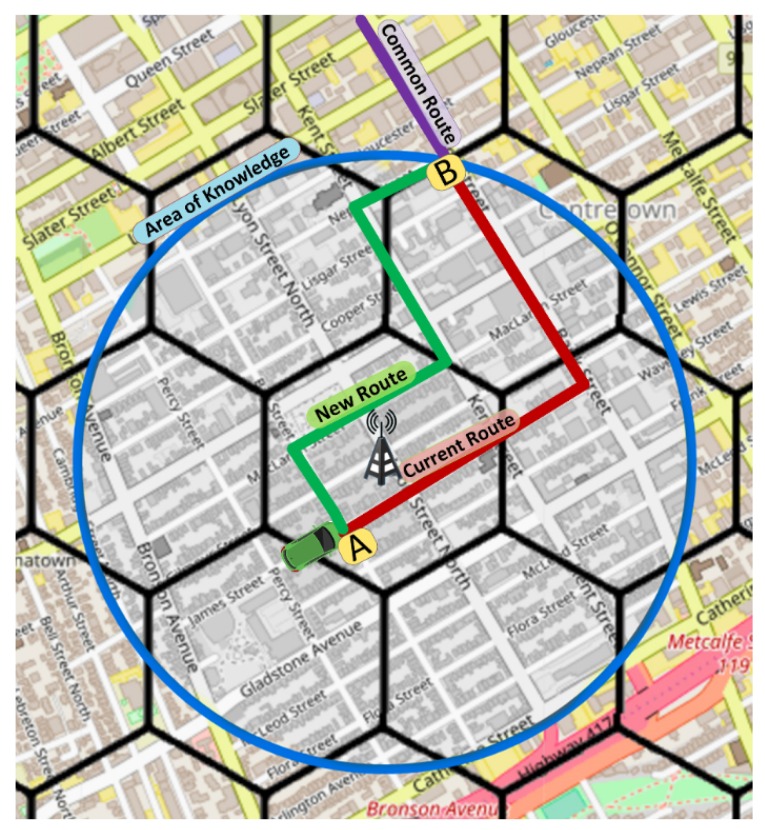
FOXS: Fast Offset Xpath Service.

Therefore, the Cloudlet computes a new route for the requesting vehicle in the scope of its AoK, that is, only using roads within the blue circle. Thus, the rerouting process does not change the part of the route outside of the AoK in purple (standard route). As we can see in Figure 7, the routing of vehicles (e.g., green car) is performed considering its current position (point **A**) until the last road in its current route that is within the AoK of the Cloudlet (point **B**).

As we can see in the Algorithm 2, the routing process begins when the RSU receives a new route request from vehicles with their information (e.g., current position, route) (Line 4, Algorithm 2). With this, the graph *G* of AoK with its congestion characteristics (in Algorithm 2 as *G*) and the variable *K* describing the maximum number of alternative routes that must be calculated.

The route weight is calculated as the sum of the weights of all the roads contained in the path. Thus, the routes with lower weight are the most requested, possibly moving the congestion from one point to another. Aiming to avoid this problem, the service computes a set alternativeRoutes of *K* alternative shortest paths as possible routes the vehicle can take (Line 8, Algorithm 2). A route from this set is probabilistically selected based on the sum of the weights (*w*) of its roads by applying the Boltzmann probability distribution [46] (Line 9, Algorithm 2).

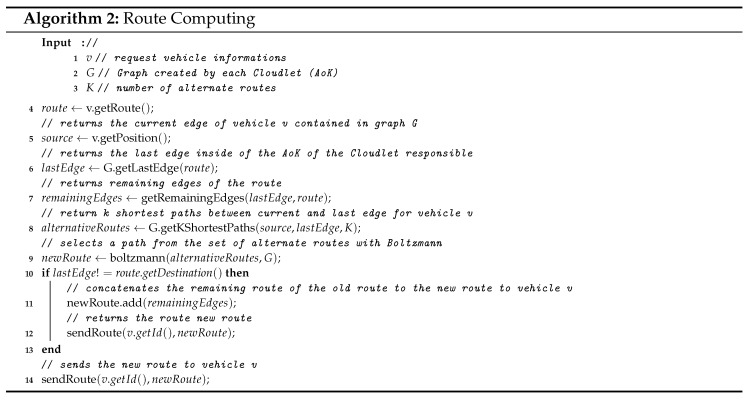



Boltzmann’s probability was chosen because it fits well with the vehicle route problem. Boltzmann probability uses the concept of temperature (e.g., route weight) to make a probabilistic choice of a route, thus preventing the algorithm to choose the same route multiple times. Thus, using the set of $R_{j}$, the vehicle traffic is balanced between roads and the general performance of FOXS is maintained. The decision rules to choose the new route are presented in equations as follows:

*J* = set of vehicles on the scenario

$R_{j}$ = set of alternative routes of the vehicle *j* (j∈J)

$r_{j}^{i}$ = route *i* of vehicle *j* (j∈J) and ($r_{j}^{i}\in R_{j}$)

$w_{j}^{i}$ = weight of route $r_{j}^{i}$

$N(w_{j}^{i})$ = normalized value of $w_{j}^{i}$ ($w_{j}^{i}\in \left[0,1\right]$) defined by Equation (1):

(1) $$\begin{array}{c} N\left(w_{j}^{i}\right)=\frac{W(r_{j}^{i})}{max\{W\left(r_{j}^{i}\right)\;|\;\forall r_{j}^{i}\in R_{j}\}}. \end{array}$$

The $K_{T}^{j}$ is the Boltzmann constant of vehicle *j* for temperature *T*, according to Equation (2):

(2) $$\begin{array}{c} K_{T}^{j}=\sum_{\begin{array}{c} i\in R_{j} \end{array}}e^{-\left(N(w_{j}^{i})/T\right)}. \end{array}$$

The $P_{T}^{j}\left(r_{j}^{i}\right)$ is the probability of choosing route *i* of vehicle *j* with the parameter of temperature *T*, according to Equation (3):

(3) $$\begin{array}{c} P_{T}^{j}\left(r_{j}^{i}\right)=\frac{1}{K_{T}^{j}}e^{-\left(N(w_{j}^{i})/T\right)}. \end{array}$$

When T→∞, all alternatives routes have the same probability of being chosen, that is, the process approaches a uniform random distribution. When T→0, the lightweight route has a high probability of being chosen.

The $E(R_{j})$ is the route chosen $\left(E\left(R_{j}\in R_{j}\right)\right)$, the choice is made according to Equation (4):

(4) $$\begin{array}{c} E\left(R_{j}\right)=\max\left\{X\times P_{T}^{j}\left(r_{j}^{i}\right)|\forall r_{j}^{i}\in R_{j},X∼\cup \left(\left[0,1\right]\right)\right\}. \end{array}$$

Once the route is selected, the system checks whether the last edge of the calculated alternative route is the destination of vehicle *v* (Line 10, Algorithm 2). If this condition is not satisfied, the new alternative route is concatenated to the remaining of the original route that lies outside the AoK of the Cloudlet that made the routing of *v* (Lines 11–12, Algorithm 2). After, the system sends a message to the vehicle with the new route.

When the requesting vehicle receives this new route, its navigation system verifies the satisfaction of the variable *route size factor*, which determines how much longer (in percent) the new route may be in comparison with the current route. If this *route size factor* does not satisfy, the vehicle maintains the current route. In this way, the system can limit the maximum size of the route, thus avoiding the increase of other traffic problems as the CO${}_{2}$ emission.

## 4. Performance Evaluation

This section shows the methodology used, the obtained results and scenario modeled to validate FOXS regarding traffic efficiency and network cost. The performance of FOXS was validated by comparing it with CHIMERA [25], PAN 1 and PAN 3 [21], Dijkstra Shortest Path (DSP) and original mobility trace, named as *BASE*.

### 4.1. Methodology

The simulations were conducted using the network simulator OMNeT++ 5 http://www.omnetpp.org. For the simulation of traffic and mobility of vehicles, we employed the SUMO 0.25.0 simulator (Simulation of Urban MObility) [47], an open source traffic simulator, which model and manipulate objects in the road scenario. For the vehicular network, was used the framework Veins 4.3 [48] that implements the IEEE 802.11p and IEEE 1609.4 DSRC/WAVE with signal attenuation model considering obstacles. To measure the CO${}_{2}$ emissions and fuel consumption, the EMIT model (describe in HBEFA http://www.hbefa.net—Handbook Emission Factors for Road Transport) integrated into SUMO was used.

We used realistic scenarios from an urban region of two big cities that suffer from traffic jam problems, Ottawa-Canada and Cologne-Germany. Such scenarios were chosen to evaluate the services in environments with different characteristics. The Ottawa scenario has a simpler structure with symmetrical streets and a smaller flow of vehicles compared to the Cologne scenario. The Cologne scenario was chosen to stress the system due to its more complex structure with fewer alternative routes and a considerably higher vehicle density. Furthermore, the network parameters for all simulations was set to 18 Mbit/s at the MAC layer and the transmission power to 2.2 mW, resulting in a coverage of approximately 300 m under a two-ray ground propagation model [48]. Table 3 shows the simulation parameters and values used in our evaluation. For all experiments, the simulations were executed 33 times with a confidence interval of 95% in the results.

All evaluated solutions follow the same operation flow (see again Figure 3). Each Cloudlet makes the traffic data gathering and road classification only in the roads contained in its coverage. Vehicles in DSP does not disseminate any information about the traffic and it uses the road length as road classification. The routing interval is set to 120 s for all solutions and three alternatives routes are used for FOXS, CHIMERA and PAN3 to obtain a fair evaluation. This number of alternative routes was chosen because it has the best results for all solutions in the evaluated scenarios. For the solutions mentioned above, we evaluated the traffic efficiency and the impact on the network, the computational resource, and the scenarios comparative analysis. For this, the evaluation was divided into two stages: Traffic Efficiency and Network and Resource Cost. For *Traffic Efficiency* evaluation, we consider the following metrics:

- *Traveled time:* the average travel time from the starting point to the destination of all vehicles;
- *Stopped time:* the average time spent stuck in traffic jams for all vehicles;
- *Average speed:* the average speed of all vehicles;
- *Traveled distance:* the average distance that all vehicles traveled;
- *Fuel consumption:* the average fuel consumption of all vehicles to traverse the whole route;
- *CO${}_{2}$ emission:* the average CO${}_{2}$ emissions for all vehicles during their trip.
- *Planning time index (PTI):* measures the reliability of the ratio of the 95% travel time to the ideal flow on the same path (e.g., a PTI of 2 means that for a 25-min trip in free flow traffic, a time of 50 min should be planned).
- *Route size Histogram:* the histogram of the number of routes by its size grouped into intervals of 500 m.
- *Cumulative Distribution Function(CDF) of the routes size:* the CDF of routes size.
- *PTI by route size:* PTI of a group of routes with similar sizes in 500 m interval.
- *PTI Utility metric:* the percentage of influence of the *PTI by route size* in the *PTI* result.

To verify the behavior of the solutions according to the route size, we present the metrics: Route size Histogram with its CDF and PTI by route size presenting the route size distribution and the relation between the PTI and the route size range. The PTI Utility metric shows the route size influence on the quality of the result in a specific scenario. The *Network and Resource Cost* were evaluated in the following metrics:

- *Transmitted messages:* the total number of messages transmitted (excluding beacon messages, which are used in all solutions);
- *Collisions per packets sent:* the percentage of collided packet per all packet sent;
- *Network delay:* the average time to spread messages to all vehicles (in milliseconds);
- *Application delay:* the average time for the application to receive the new route when requested, with the service response time and retransmission time when necessary (in milliseconds);
- *New route accepted:* the average of new route accepted per vehicle in simulation;
- *Cloudlet routes computed:* the average of routes computed per Cloudlet;
- *Cloudlet computation heat-map:* representing the amount of routing executed by regions.

### 4.2. *Ottawa* Scenario

**Figure 8:**
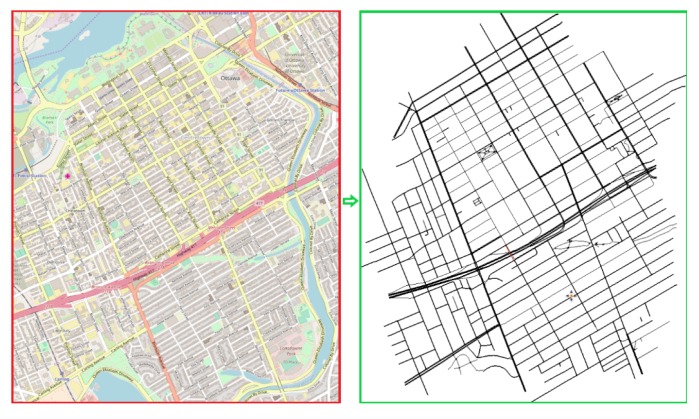
Topology of the Ottawa Scenario.

Ottawa scenario (Figure 8) represents a downtown region and was obtained from OpenStreetMap http://www.openstreetmap.org/, by possessing an area of 8 km${}^{2}$. The scenario has 409.42 km of total road length and 2.200 vehicles inserted during the simulation representing congested traffic.

#### 4.2.1. Impact of Traffic Efficiency

**Figure 9:**
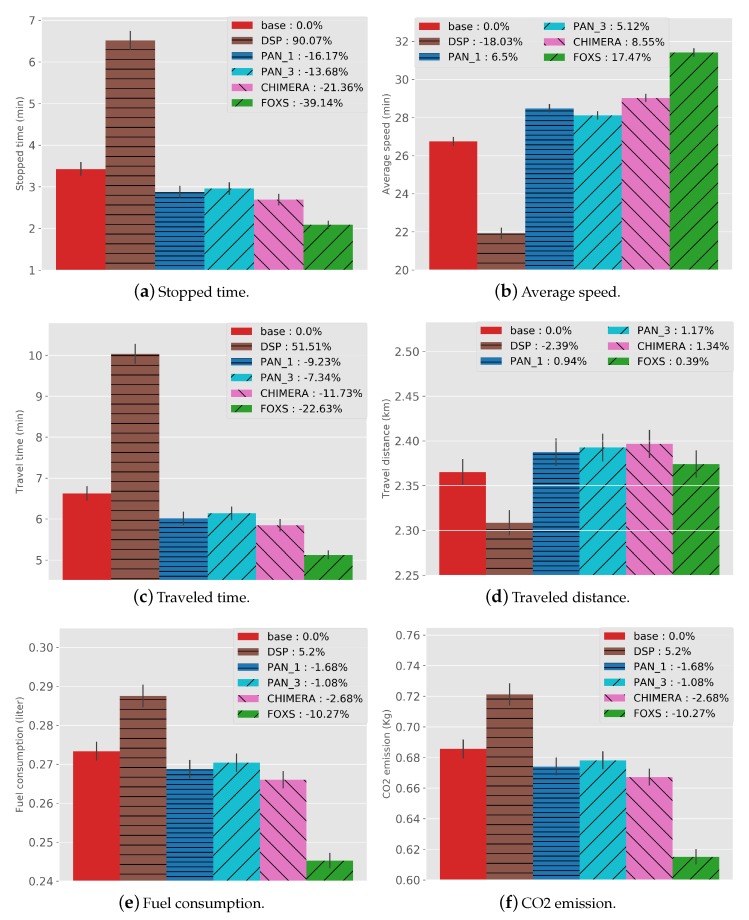
Traffic Efficiency Results—Ottawa Scenario.

Figure 9 presents values and *BASE* related percentage of all metrics for the implemented solutions concerning traffic efficiency. The solutions PAN1, PAN3, CHIMERA and, FOXS reduced the stopped time (Figure 9a) in 16.17%, 13.68%, 21.36% and, 39.14% respectively in relation to the *BASE* solution. These results reflect a higher average speed, as presented in Figure 9b. FOXS has a better performance than all evaluated solutions, increasing the average speed by 17.47% because the FOXS uses a better road classification and the probabilistic mechanism to choose one of K alternatives routes. Note also that the DSP had its stopped time increased in 90% and the average speed reduced in 18% compared to the *BASE*. This happens because DSP only calculates the shortest path for all vehicles, moving the vehicles to the same road. Consequently, creating a new congestion point unlike the other evaluated solutions, that calculates a new route when necessary and based on the road conditions. Considering the average speed metric, PAN1 (6.5%) has better results than PAN3 (5.1%). This happens because PAN3 calculates three alternatives routes and choose one at random. Thus, the second and third bests routes may have different sizes compared to the best route due to the geography of the city map. This not happens in FOXS, because the alternative route is chosen in a probability way reducing the chance of choosing a wrong route and the use of the route size factor. Similar behavior can be observed when we look at the travel time presented in Figure 9c. FOXS manages better the urban traffic reducing in 22.63% the travel time, twice times less than CHIMERA, which reduces 11.73% compared to *BASE*.

Analyzing the traveled distance (Figure 9d), only the DSP decreases the distance compared to the *BASE* (2.39%), because it always chooses the shortest route to destination besides the other solutions that calculate the new route based on the road condition. Considering FOXS, alternative routes, on average, are not 0.39% longer than the original ones. These results can be explained by the fact that FOXS employs a parameter that controls how much longer alternative routes can be. Figure 9f,e shows the $CO_{2}$ emission and fuel consumption respectively. The traveled time, the stopped time and the traveled distance have a direct impact on these metrics. The results present that FOXS reduces three times the $CO_{2}$ emission and fuel consumption (10.27%) compared with the CHIMERA that reduces 2.68%.

**Figure 10:**
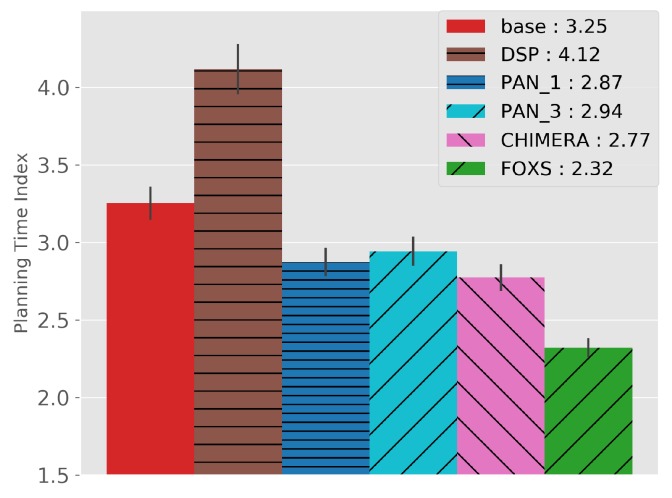
Planning Time Index (PTI)—Ottawa Scenario.

**Figure 11:**
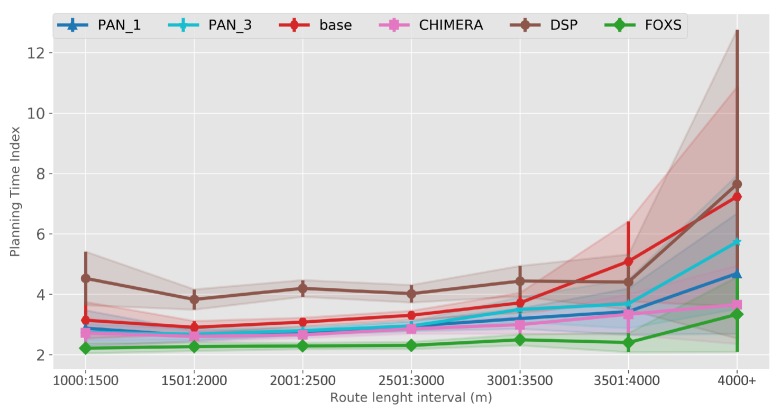
PTI by route size—Ottawa Scenario.

**Figure 12:**
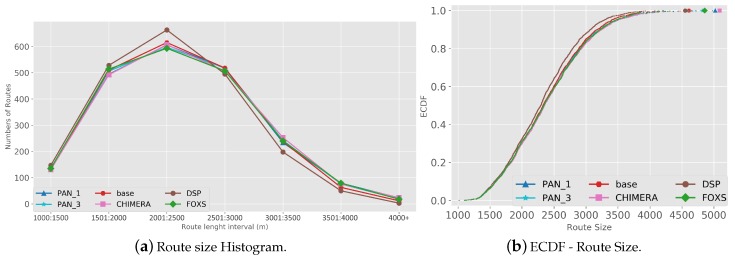
Route Size Analise—Ottawa Scenario.

**Figure 13:**
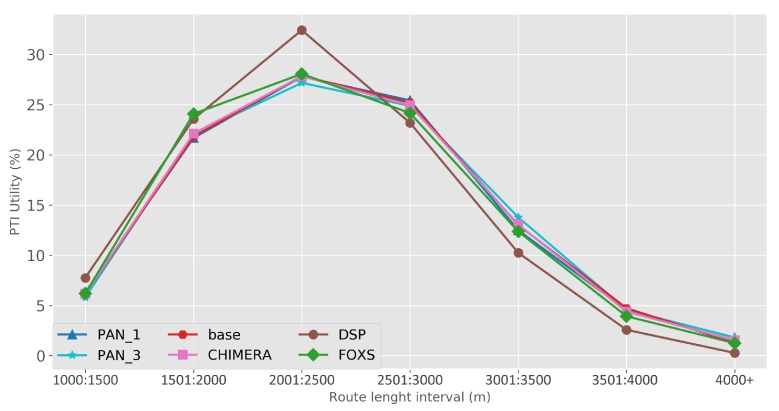
PTI Utility metric—Ottawa Scenario.

The quality of the congestion control of the solutions evaluated using the PTI is presented in Figure 10. Among the evaluated solutions, FOXS obtained the lowest PTI index (2.32), being 19% lower than the CHIMERA. To verify which solutions work better according to the route size, Figure 11 shows the PTI for 500 m intervals. We verified that the FOXS behaved stably with routes up to 4 km having a small increase in the PTI index for larger routes. In Figure 12 is presented the route size histogram and route size CDF. The DSP has more shortest routes because the solution always chooses the smallest route and the CHIMERA had the longest routes than FOXS, PAN1 and PAN3 (see Figure 12a). Note, for all solutions 80% of routes is shorter than 2700 m (see Figure 12b). The PTI Utility metric (see Figure 13) shows that route with the size between 1.500 m and 3.000 m is the most congested, representing 70% of total routes in the scenario.

#### 4.2.2. Impact of Network and Resource Cost

**Figure 14:**
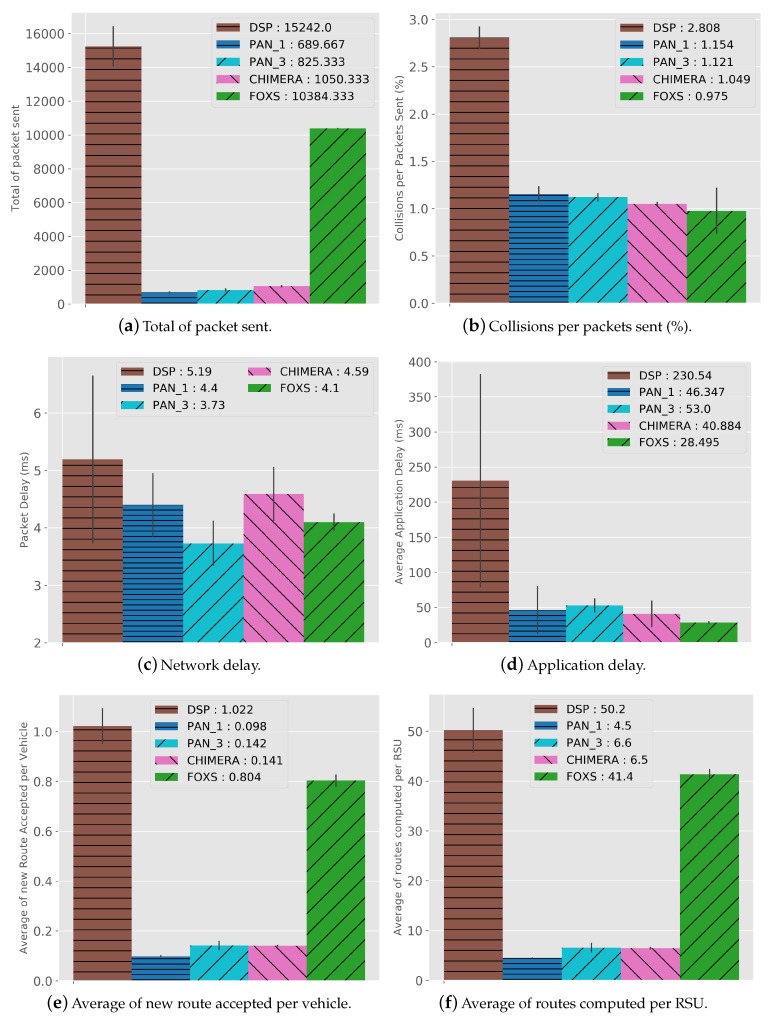
Network Cost Results—Ottawa Scenario.

Analyzing the network metrics, in Figure 14 we can see the results to the defined metrics. As seen in the traffics results, FOXS obtained better performance in traffic management. However, FOXS generates a higher amount of network messages (10384) compared to the CHIMERA, PAN1 and PAN3 (Figure 14a). This happens due to the FOXS congestion detection mechanism be more effective. Consequently, increasing the average routing performed by the vehicles in the system (routes received and altered by the vehicles) thus improving urban traffic. Figure 14e shows that the FOXS obtained an average of 0.8 routing per vehicle compared to 0.14 in CHIMERA thus, exchanger fewer control messages with the RSU. Note that a high number of routing performed by vehicles (e.g., DSP with 1.02) does not imply in a better result in the traffic (see Figure 9). Figure 14f presents the average of routes computed per RSU in the scenario during the simulation. These values are directly related to the average of new route accepted per vehicle.

Despite the higher volume of network messages generated by FOXS, as described above, FOXS handle the urban traffic better distributing the vehicles across the map avoiding the creation of new traffic jam. Consequently, the number of network collisions (0.9%) is lower among the solutions (see Figure 14b). However, the amount of congestion generated in the scenario who use the DSP (see Figure 9), leads to a higher number of packet collisions (2.8% of total packets). The application Delay (Figure 14d) is influenced by packet collisions and by the number of routing executed per RSU. Because when the routing request period arrives, several vehicles may request a new route at the same time. Thus, overloading the network and the RSU, that for each requesting vehicle, has to compute a new route and create a message to send the new route to vehicles. FOXS has a packet loss of 0.5% of the total messages sent.

**Figure 15:**
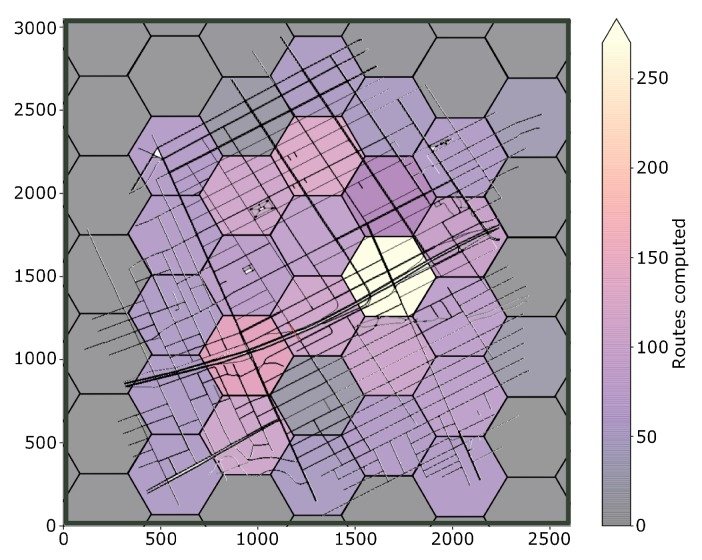
Routes computed per region-Ottawa Scenario.

In Figure 15 is presented the heat-map representing the amount of routing executed by regions (Cloudlet-RSU). With this analysis, it is possible to implement, for each region, Cloudlets with computing and communication power based on the demand of users. Thus, saving equipment cost. Also, a load-balance can be made sharing resource to more occupied Cloudlet. Specifically for FOXS, this information can be used to adjust its settings.

### 4.3. *Cologne* Scenario

**Figure 16:**
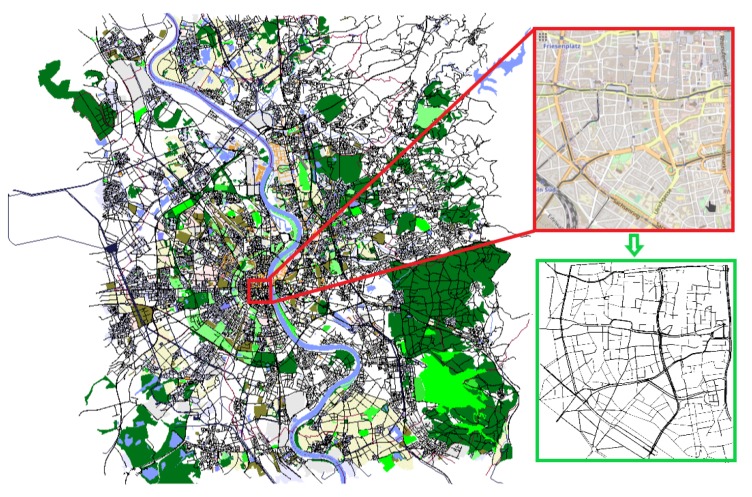
Topology of the Cologne Scenario.

Cologne scenario is a sub-part of a TAPAS Cologne scenario [49] (presented in Figure 16) that includes the 24 h of the mobility trace of the vehicles, obtained through real monitoring of the city traffic. Due to the large scale of the TAPAS scenario, we used a more critical traffic time (7 am to 8 am) and a sub-area of 4.5 km${}^{2}$ of the Cologne downtown. This area possesses the greatest density and flow of vehicles, thus maintaining the representation of the scenario for our analysis. This scenario has approximately 14.000 vehicles inserted during the simulation and roads with 243 km of total length.

#### 4.3.1. Traffic Efficiency Evaluation

**Figure 17:**
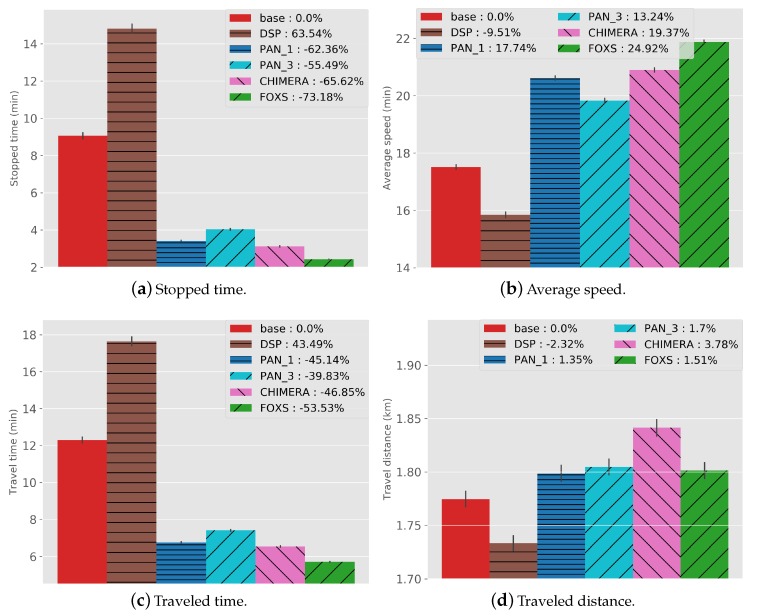

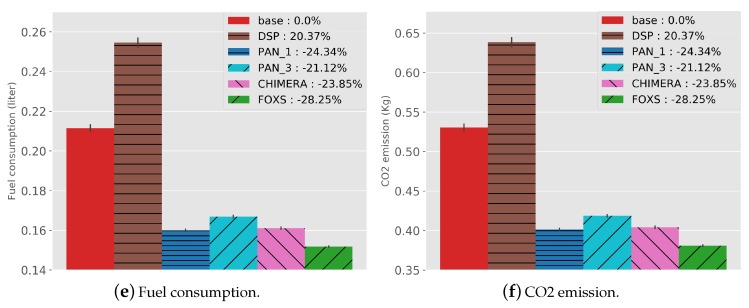
Traffic Efficiency Results—Cologne Scenario.

Figure 17 shows the values and its percentage in relation to the *BASE* solution. In this scenario, all the solutions evaluated had a similar behavior compared to the Ottawa scenario. However, the main difference between them was reduced, due to a greater vehicle density in the Cologne scenario (3.111 vehicles per km${}^{2}$ in one hour) when compared to the Ottawa scenario (245 vehicles per km${}^{2}$ in one hour). Thus, increasing the demand for alternative routes and requiring a more effective solution.

As expected, DSP reduced the distance traveled (2.32%) and the PAN1 and FOXS had the lowest route increase with 1.35% and 1.53% respectively (Figure 17d). Note that CHIMERA increases the route size in 3.78% related to *BASE* and 2 times related to FOXS. This is because CHIMERA does not have a mechanism to evaluate the size of the route as FOXS. The Stopped time (Figure 17a) was reduced for PAN1 (62.36%), PAN3 (55.49%), CHIMERA (65.62%), FOXS (73.18%) and the Average speed (Figure 17b) was increase PAN1 (17.74%), PAN3 (13.24%), CHIMERA (19.37%) and FOXS (24.92%) in relation to *BASE*. Note there is a high difference between FOXS, PAN1, PAN3 and CHIMERA to *BASE*. This is caused by the large volume of vehicles on the scenario and by the great impact generated during the choice of alternative routes since the roads have a quite different size. However, FOXS chooses better routes than all solutions because it considers the size of the new route beyond the route classification. The profit reached by FOXS in previous metrics is reflected in the improvement of the fuel consumption (reducing 28.25%, Figure 17e), of the CO_2_ emission (reducing 28.25%, Figure 17f) and of the travel time (reducing 53.53%, Figure 17c). About the PAN1 and PAN3, which differ only by the alternative routes, it was observed that for a real scenario, the random choice of a set of best routes does not provide a good result as presented in Figure 17.

**Figure 18:**
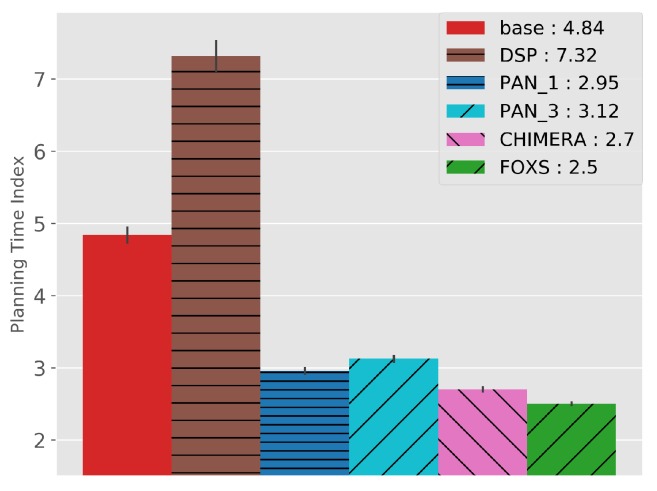
Planning Time Index (PTI)—Cologne Scenario.

**Figure 19:**
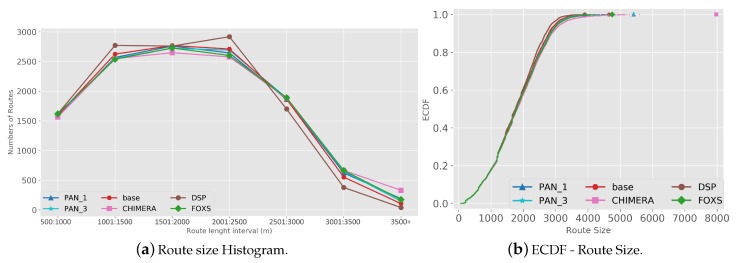
Route Size Analise—Cologne Scenario.

**Figure 20:**
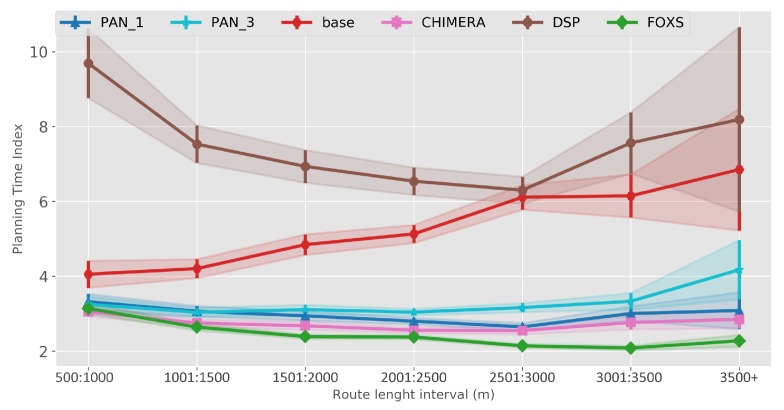
PTI by route size—Cologne Scenario.

**Figure 21:**
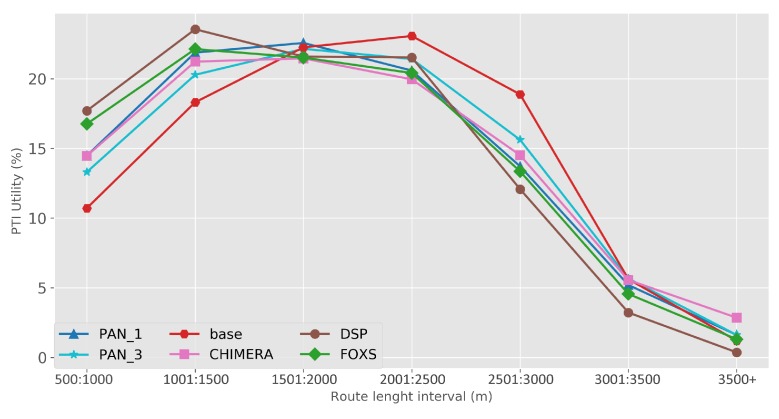
PTI Utility metric—Cologne Scenario.

The metric PTI was reduced in 49% by FOXS, in 44% by CHIMERA and in 39% by PAN1 compared to *BASE* (Figure 18) showing the efficiency of these solutions. These results show that FOXS is able to better handle the city traffic, corroborating with the results obtained in Figure 17. As we can see in Figure 19, Cologne scenario has short routes where 80% of routes are shorten than 2.500 m (see Figure 19b) and approximately 60% of the routes its between 1.500 m and 2.500 m (see Figure 19a). Short routes hamper a good response of routing solutions since the set of alternative routes will be smaller. Introducing the PTI for each size range of routes showed in Figure 20, the FOXS had decreases the PTI when the route increases. These two graphs show that FOXS is better on larger routes in this specific scenario. The analyzes of the PTI Utility metric (see Figure 21) presents that 75% of more congested routes has a size between 1.000 m and 2.500 m.

#### 4.3.2. Network and Resource Cost Evaluation

**Figure 22:**
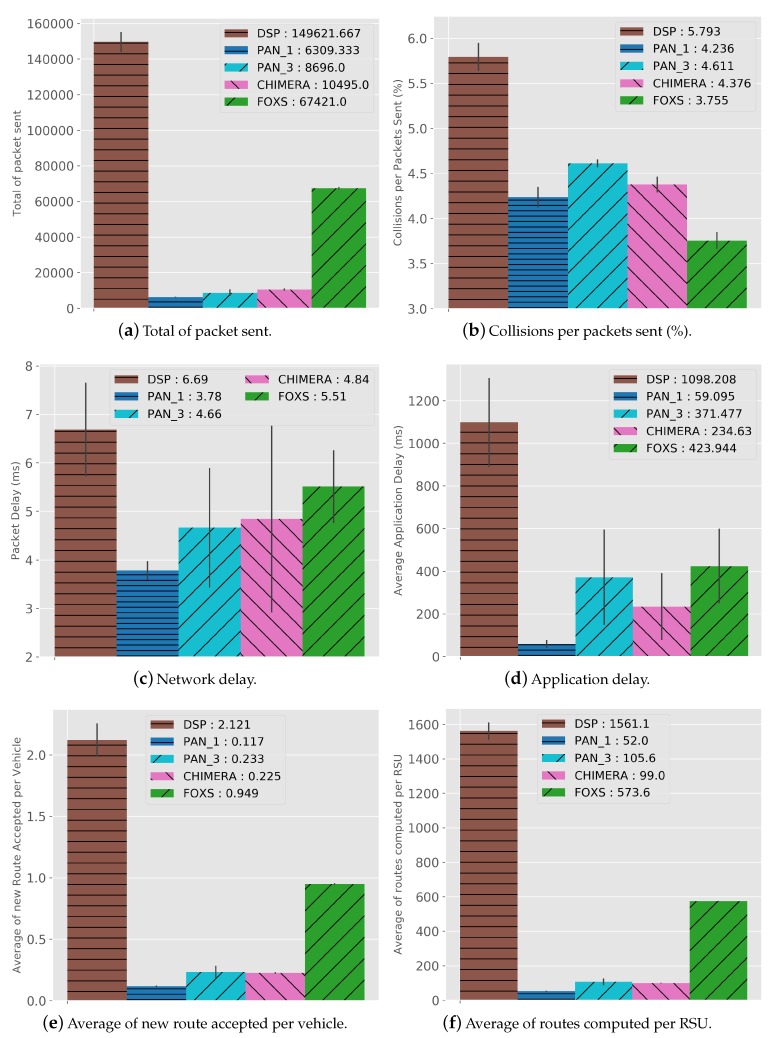
Network Cost Results—Cologne Scenario.

**Figure 23:**
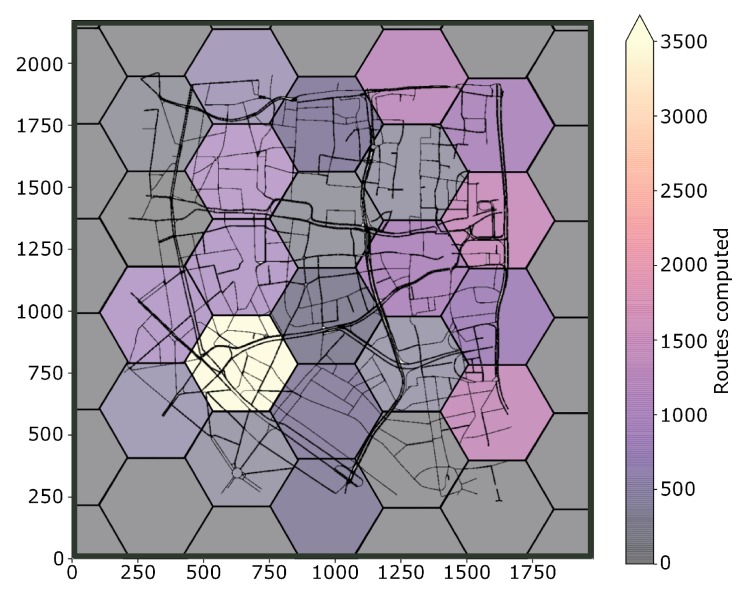
Routes computed per region—Cologne Scenario.

Figure 22a shows the total number of messages during the simulation. Note that FOXS sends more messages comparing to the CHIMERA, PAN1 and PAN3. However, FOXS has better average of the routes attributed to vehicles (see Figure 22e) compared to PAN1 (0.117 reroute per vehicle), PAN3 (0.233 reroute per vehicle) and CHIMERA (0.225 reroute per vehicle) (Figure 22e). Thus, the greater number of routed vehicles in the right way to increase to the quality of the city’s traffic. Note that the high number of routes accepted does not always produce a good result as seen in DSP (2.121 reroute per vehicle). The average of routes computed by each RSU-Cloudlet is presented in Figure 22f. FOXS had the average of 573.6 routes computed and PAN1, PAN3 and CHIMERA had the average of 52, 105.6 and 99 routes computed respectively. The high number of routes computed by FOXS not becomes a problem because of the use of the Fog paradigm, which distributes the calculation of the routes by regions in several Cloudlets. Figure 23 geographically shows these route calculation distribution of FOXS on the map exposing the areas with the most demand for Cloudlets.

The behavior of the number of packet collisions (Figure 22b) shows that FOXS has a result of approximately 11.5% better than PAN1. Despite the large volume of packets generated by FOXS, the network delay (see Figure 22c) did not have a significant difference to CHIMERA and PAN3. This was due to the messaging scheduling mechanisms and effective routing by distributing the vehicles in the scenario. Thus, avoiding the concentration of vehicles in a region competing with the network channel. The application delay (Figure 22d) is influenced by the number of routes computed and the others network metrics. We can see that although FOXS has a large number of computed routes and packet sent, the number of packets collisions was the smallest among the solutions. Thus, its application delay had a value similar to all evaluated solutions. Finally, such results show that FOXS is better suited to handle traffic jams in the evaluated scenarios.

### 4.4. Scenarios Comparative Analysis

Considering the different characteristics of the evaluated scenarios, a comparative evaluation of these scenarios was also made.

The Ottawa scenario possesses a more symmetrical structure and lower vehicle flow when compared to Cologne scenario. Thus, allowing FOXS to have a higher gain when compared to other solutions. We can highlight the difference between FOXS and CHIMERA, the second most efficient solution. The difference between these solutions when considering PTI metric was 19% in the Ottawa scenario and 8% in the Cologne scenario. We can also observe such behavior in all traffic related metrics, such as Stopped time, where the difference is 20% in the Ottawa scenario and approximately 9% in the Cologne scenario.

Regarding network metrics, although in the Cologne scenario there is an increase in the number of vehicles on the network, the performance of FOXS, when compared to other solutions, is less compromised as we can see in the metric Collisions per packets sent. This is due to the effectiveness of the messaging scheduling mechanisms that FOXS implements. When considering the metric Average of New Route accepted per vehicle, FOXS increased it from 80% in the Ottawa scenario to 95% in the Cologne scenario. Therefore, these results corroborate the dynamics adaptation of FOXS in relation to the characteristics of the scenarios where FOXS achieves a good result outperforms the others solutions evaluated.

## 5. Conclusions

This article presented FOXS, a traffic service to route suggestion that employs FOG computing. The use of Fog computing paradigm introduces several benefits to the FOXS such as computational distribution, providing the processing load balancing and proximity of computational resources to the end-users, decreasing the response time and the network bandwidth usage. FOXS design was divided into three stages: (i) Data Gathering, where all data are collected, (ii) Data Processing, where the gathered data is transformed in relevant information to the service and (iii) Service Delivery, where the route suggestion is computed and forwarded to users. Several experiments in two different urban scenarios were executed in order to show the efficiency of the proposed traffic service. These experiments showed the reduction of the drawbacks generated by the traffic congestion in the stopped time (reduction of 70%), the CO${}_{2}$ emissions (reduction of 29%) and the planning time index (reduction of 49%). Related to network metrics the packet collisions and the application delay was reduced in 11.5% and 30% compared to others solutions. As future work, we are planning to execute experiments considering different scenarios and the use of other communication technologies such as LTE or 5G. Besides, the proposed service could be implemented considering a Cloud computing interaction to increase the route suggestion efficiency using historical information about the traffic jam and drivers characteristics. We will also evaluate the performance of these solutions with partial coverage of the scenario and other techniques regarding road classification.

## Figures and Tables

**Table 1:** Comparative of related solutions.

Work	Communication Architecture	Processing Architecture	Track Shift Avoid	Route Size Check	Route Area	RSU Interaction	Message Management
**PAN (i) [21]**	V2I	Centralized	✗	✗	All map	✗	✗
**PAN (ii) [21]**	V2I	Centralized	Random	✗	All map	✗	✗
**CHIMERA [25]**	V2I	Distributed RSU	Probabilistic	✗	RSU corverage	✔	✗
**EcoTrec [34]**	V2V/V2I	Distributed Vehicles	Random	✗	All map	✗	✗
**INCIDEnT [33]**	V2V	Distributed Vehicles	✗	✗	Neighborhood	✗	✗
**ECODE [26]**	V2V/V2I	Distributed RSU	✗	✗	Neighborhood	✔	✔
**NRR [37]**	V2I	step1: distributed iTL step2: centralized	✗	✗	All map	✗	✗
**SAINT [38]**	V2I	Centralized Cloud	Traffic balancing	✗	All map	✗	✗
**FOXS**	V2V/V2I	Distributed Fog	Probabilistic	✔	Defined region	✔	✔

**Table 2:** Data exchange by DSRC/WAVE communication channel.

DSRC/WAVE Channel	Vehicle Messages	Cloudlet Messages
**Control Channel(CCH)** **Beacon**	–Speed –Position –Time spent to move on each road –Current route	–Position –Region route interval –List of congested roads
**Service Channel(SCH)** **Data**	Request route	Response new route

**Table 3:** Simulation parameters.

Parameters	Values
Transmission power	2.2 mW
Communication range	300 m
Bit rate	18 Mbit/s
Beacons	4 s
Alternatives routes (k)	3
Confidence interval	95%
AoK	3000 m
Route size factor	25%
Interval to request new route	120 s
